# Assessing data linkage quality in cohort studies

**DOI:** 10.1080/03014460.2020.1742379

**Published:** 2020-05-20

**Authors:** Katie Harron, James C. Doidge, Harvey Goldstein

**Affiliations:** a Department of Population, Practice and Policy, UCL Great Ormond Street Institute of Child Health, London, UK;; b Intensive Care National Audit and Research Centre (ICNARC), London, UK;; c School of Education, University of Bristol, Bristol, UK

**Keywords:** Cohort studies, data linkage, measurement error, administrative data, selection bias

## Abstract

**Background:** Linkage of administrative data sources provides an efficient means of collecting detailed data on how individuals interact with cross-sectoral services, society, and the environment. These data can be used to supplement conventional cohort studies, or to create population-level electronic cohorts generated solely from administrative data. However, errors occurring during linkage (false matches/missed matches) can lead to bias in results from linked data.

**Aim:** This paper provides guidance on evaluating linkage quality in cohort studies.

**Methods:** We provide an overview of methods for linkage, describe mechanisms by which linkage error can introduce bias, and draw on real-world examples to demonstrate methods for evaluating linkage quality.

**Results:** Methods for evaluating linkage quality described in this paper provide guidance on (i) estimating linkage error rates, (ii) understanding the mechanisms by which linkage error might bias results, and (iii) information that should be shared between data providers, linkers and users, so that approaches to handling linkage error in analysis can be implemented.

**Conclusion:** Linked administrative data can enhance conventional cohorts and offers the ability to answer questions that require large sample sizes or hard-to-reach populations. Care needs to be taken to evaluate linkage quality in order to provide robust results.

## Introduction

Data linkage is an important tool for generating longitudinal data that can be used to understand the development and causes of variation in outcomes across the life course (Chamberlayne et al. 1998; Jutte et al. 2011). Linkage of administrative data sources can provide an efficient means of collecting detailed data on cross-sectoral services (e.g. health, social care and education), society, and the environment, as well as augmenting direct data collection through linkage with biological samples, social media and other digital sources. Linked data can be used to supplement conventional cohort studies or to create population-level electronic cohorts generated entirely from administrative data (Hockley et al. 2008; Ford et al. 2009; Doiron et al. 2013; Ali et al. 2019; Downs et al. 2019). Such administrative data cohorts offer the ability to answer questions that require large sample sizes or detailed data on hard-to-reach populations, and to generate evidence with a high level of external validity and applicability for policy-making (Chiu et al. 2016). There is increasing interest in using these two models of data collection in conjunction, combining population-level administrative data with detailed attribute data collected directly from participants, in order to provide a deeper insight into what determines our health (Boyd et al. 2019).

A major challenge to generating reliable linked data that are fit for purpose is the availability of accurate identifiers that can be used to link the same person across multiple data sources (Gilbert et al. 2018). Ideally, a single unique identifier such as National Health Service (NHS) number or National Insurance number would be recorded accurately in all datasets, enabling a straightforward linkage between sources. In practice, such an identifier is rarely available, particularly when linking data across sectors (e.g. health to education) and nearly always involves some degree of error or missing data (Ludvigsson et al. 2009). Therefore, linkage often depends on the use of non-unique identifiers such as name, postcode and date of birth. Such a set of identifiers can provide, in combination, sufficient discrimination between individuals to facilitate linkage but can also lead to linkage error and uncertainty.

Due to the limitations of available identifiers, errors can occur during linkage and manifest as false matches (where records belonging to different individuals are linked together) or missed matches (where records belonging to the same individual are not linked) (Table 1). False matches occur when different individuals share the same identifiers (e.g. through system errors that have assigned the same NHS number to a pair of twins), or where identifiers are not sufficiently discriminative (e.g. where two household members share the same surname, gender and postcode). Missed matches can occur due to recording errors (e.g. misspelt names), genuine changes over time (e.g. when an individual moves to a new postcode area), or where missing or insufficiently distinguishing identifiers prevent a link from being made. The level of linkage error is dependent on the quality and completeness of the identifying data available within a dataset, and can occur irrespective of the linkage methods employed (Doidge and Harron 2018). However, careful data cleaning and linkage design can help reduce the likelihood of errors, and linkage strategies can be designed to minimise false matches or missed matches (or to strike a balance between the two), depending on the aims of research (Doidge and Harron 2018).

**Table 1.** Linkage accuracy.

	True match status	
	Match (pair from same individual)	Non-match (pair from different individuals)
Assigned link status		
Link	True match *a*	False match *b*
Non-link	Missed match *c*	True non-match *d*

Sensitivity (or recall) = *a*/(*a* +* c*); specificity = *d*/(*b* +* d*); positive predictive value (or precision) = *a*/(*a* +* b*); negative predictive value = *d*/(*c* +* d*).

Despite advances in linkage methods and improvements in data quality over time, some level of linkage error or uncertainty is almost always inevitable in linkage of administrative datasets that were not collected primarily for research (Harron et al. 2017). There is a large amount of literature demonstrating that even low levels of linkage errors can have important implications for analysis, and that if these errors are not addressed, substantial bias may be introduced into results derived from linked data (Bohensky et al. 2010; Lariscy 2011). The impact of such errors depends not only on the error rates, but also on the distribution of errors in relation to analysis variables. It also depends on the structure of the linkage design and the analysis in question. Tolerable levels of error therefore need to be considered on a case by case basis, taking all these factors, and the implications for inferences, into account (Doidge and Harron 2019).

This paper provides an overview of linkage error as it relates to cohort studies and provides guidance on how to assess linkage quality and its implications for analysis of linked cohort data. We first describe relevant methods in general and then discuss illustrative results from published studies.

## Methods

### Methods for data linkage

Traditional linkage methods fall into two broad and overlapping classes: deterministic (rule-based) algorithms and probabilistic linkage techniques involving “match weights.” Deterministic methods typically make use of a set of pre-specified rules for classifying pairs of records as belonging to the same individual or not. For example in national hospital data in England (Hospital Episode Statistics), admissions for the same individual are linked over time using a three-step algorithm involving NHS number, date of birth, postcode and sex (Hagger-Johnson et al. 2015). More complex deterministic methods may incorporate the use of partial identifiers (e.g. postcode prefix or first letter of surname), similarity scores, or transposition of elements of date of birth. However, as the number of available identifiers increases, the number of variations can become unmanageable using deterministic rules.

Probabilistic linkage methods work by assigning a weight or score to represent the likelihood that two records belong to the same individual. In effect, this results in a ranking of all possible deterministic rules for a set of available identifiers (Doidge and Harron 2018). We note that what is typically classed as “probabilistic” linkage is effectively a sophisticated version of deterministic linkage, since match weights will ultimately be used to define a deterministic classification of records using one or more thresholds. There are a number of methods for deriving such probabilistic match weights or scores but most are based on the Fellegi-Sunter algorithm, which uses the conditional probability of agreement on an identifier, given whether two records belong to the same individual or not (Fellegi and Sunter 1969; Sayers et al. 2016). However, this approach relies on a number of assumptions (Goldstein et al. 2017). It also involves an initial estimation of conditional probabilities either using training data (where the true match status is known for a sample of records) or using statistical techniques such as the EM algorithm.

Machine learning approaches to linkage are also being developed based on computationally predicting the likelihood of records belonging to the same individual (Elfeky et al. 2003; Christen and Goiser 2007; Pita et al. 2018). However, suitable training datasets are rarely available to support these methods (Christen and Pudjijono 2009). An alternative unsupervised method, employing a scaling algorithm originating from correspondence analysis, has been developed to overcome this problem but is yet to be implemented outside of simulation studies (Goldstein et al. 2017). The scaling algorithm assigns scores to discrete categories or degrees of agreement/disagreement based upon minimisation of a suitable loss function.

Identifiers used for linkage need not be personal in nature; any variable that is represented in both records, and even different variables that are correlated (e.g. the date of finishing one service and the date of starting another), can be used to inform linkage (Lawson et al. 2013; Li et al. 2015). Both deterministic and probabilistic techniques can be tailored towards minimising one type of error or the other, but deterministic techniques are often designed to minimise potential for false links, and the greater flexibility of probabilistic techniques can often support detection of more true matches (i.e. fewer missed matches) without accepting higher rates of false matches (Hagger-Johnson et al. 2017). Even though probabilistic linkage techniques can often perform better, their complexity and computational requirements mean that deterministic linkage is often preferred, especially with data that include smaller numbers of high-quality identifiers.

### Methods for using linked data in cohort studies

Use of linked data in cohort studies also falls into two broad classes: (i) supplementation of primary data collection in conventional cohorts with linked data that has been collected for other purposes (often population-level administrative and registry data), and (ii) construction of electronic cohorts solely from secondary data sources, usually retrospectively and relying on de-identification in place of consent. Data linkage is supporting new models of efficient cohort research such as UK Biobank, in which large-scale collection of biological specimens provides the main source of primary data for a cohort, with most other data provided through routine linkage to population-level datasets (Davis et al. 2019).

There are important differences between cohorts based on primary data collection supplemented with linked data, and cohorts derived from linked data alone. These differences relate to how participants are identified for inclusion in analysis, and the potential for linkage and linkage error to influence this process. In conventional cohorts, participants are a subset of the population that is clearly defined as those consenting to participate. Each participant is represented once within the cohort and followed up over time. For any supplementary linked data, it is apparent which participants have linked data and which do not (although not always which participants *should* have linked data). Most administrative data cohorts are created from event-based datasets (hospital admissions, etc.) and even when only one such dataset is used, the records within it have to be linked internally to create a longitudinal record for each individual (Herbert et al. 2015). Even registries (births, deaths, notifiable diseases, etc.) that aim to record people or events only once can contain varying levels of duplication depending on the systems used to collect the data. Thus, when creating a cohort from administrative data sources, linkage and linkage error have the potential to affect the specification of the cohort itself.

### Methods for assessing linkage quality in cohort studies

There are a number of mechanisms by which linkage errors can bias analyses based on linked data. In order to understand these mechanisms, we first need to understand the structure of the linkage to be performed and the purpose of the linkage. In practice, it is likely that a given study will aim to link data from multiple (>2) files. This can either be done using one primary “spine” dataset (e.g. a cohort) and linking each new file to the spine, or by sequentially linking pairs of files together. For simplicity, we start by considering how data from one or two files can be combined and analysed, and represent these scenarios using Venn diagrams (for a detailed list, see (Doidge and Harron 2019)). Even when linking more than two files, this pairwise approach can still be useful for considering the implications of linkage error. The target cohort is typically defined by only one or two files, with remaining files linked to that cohort. If linkage between the remaining files is also conducted, then indirect links may be formed with the cohort (e.g. a link from a record in file A to a record in file B that is linked to the cohort creates an indirect link between file A and the cohort), but how linkage errors manifest is unaffected by whether the links are made directly or indirectly. Perhaps the most common and relevant to cohort studies are the “Master,” “Nested” and “Intersection” structures (Table 2).

**Table 2.** Common linkage structures for combining data from two sources, one of which is a cohort study.*

Linkage structure	Example	Purpose	Implications of a missed match	Implications of a false match
“Intersection”	The large circle represents a national dataset containing records of school attainment (e.g. the National Pupil Database in England) and the small circle represents a cohort study. The school database will include records for some individuals who are not cohort participants. Not all cohort participants may be captured in the school database (e.g. those who moved out of the country before starting school). Analysis is restricted to cohort participants with a linked school record.	To define the study population.	Exclusion from the study sample and potential selection bias (cohort participants without linked school records are excluded).	Measurement error or misclassification in any school variables obtained through linkage.**
“Master”	The large circle represents a cohort study and the small circle represents a disease registry linkage with the disease registry will be meaningfully interpreted as a cohort participant having the disease. The shaded area indicates that data from all cohort participants will be analysed.	To define exposure/outcome.	Misclassification of disease status, i.e. a cohort participant is erroneously classified as being disease-free.	Misclassification of disease status (if a cohort participant who does not have the disease is linked with the disease registry).***
“Nested”	The large circle represents birth registration data and the small circle represents a cohort study. All cohort participants are expected to have a birth registration record, but the birth registration data will include some individuals who are not cohort participants. The cohort defines the analysis sample; participants who are linked with a birth registration data have further information on variables of interest.	To add further information on variables of interest.	Missing data: no birth registration variables will be available for cohort participants without a linked record.	Measurement error or misclassification in any birth registration variables obtained through linkage.**

Shaded areas represent the study sample for a particular research question. The relative size of the circles does not matter. We assume that the cohort sample is uniquely identified prior to linkage (i.e. there is only one record per participant) but that the linked data (e.g. administrative data) may contain multiple records per person.

* Although we have used cohort studies as an example here, this is not a general requirement for these linkage structures. **If a false match is made to a record that (by chance) holds the same values of analysis variables as the true match, measurement error or misclassification would not occur. ***If a cohort participant who does have the disease is linked with the wrong registry record, this could lead to measurement error or misclassification in any other variables captured about the disease (e.g. stage or type of cancer).

There are three main reasons to perform linkage: (i) to define a study sample; (ii) to define a variable of interest when the value of that variable is inferred through linkage itself (e.g. linking with a disease registry to infer disease status); or (iii) to provide information about additional variables of interest obtained through linkage. Table 2 outlines the implications of missed matches and false matches for these three purposes. In general, if the purpose is to define a study population, then linkage error can lead to erroneous exclusion or inclusion from the study population (i.e. through missed or false matches where linkage provides information on inclusion/exclusion criteria). Such errors can lead to bias, or loss of statistical power and incorrect measures of precision. If the purpose is to define a variable of interest, or to provide information on additional variables, false matches can lead to misclassification or measurement error in any of the variables captured through linkage (i.e. if the wrong records are linked together). In all cases, linkage errors can result in bias or missing data.

In administrative data cohorts, linkage error can also result in double-counting (when one individual’s records are counted multiple times, due to missed matches), or undercounting (when records for multiple individuals are counted as one, due to false matches). A detailed discussion of how linkage error can impact on results from linked data is provided elsewhere (Doidge and Harron 2019).

Once we have established how linkage error could manifest in a given analysis, the next step is to try to estimate the extent of error. It is also important to estimate the distribution of linkage errors with respect to variables of interest: when linkage errors are not distributed randomly, i.e. are more likely to occur in one subgroup than another, results can be substantially biased, even when overall error rates are low. There are many examples of differential linkage quality in the literature (Bohensky et al. 2010; Bohensky 2015). Data quality (and therefore linkage quality) can be related to ethnicity, age, socio-economic status, or health status, and small subgroups of individuals (who may be the most interesting for analysis) are often those most affected by linkage error. Understanding the distribution of linkage errors is therefore vital for evaluating potential bias due to linkage error.

Estimation of linkage error rates is useful to both data linkers and analysts of linked data. Firstly, information about the likely error rates associated with different patterns of agreement can help inform decisions about how to classify links (e.g. the deterministic rules or match weight thresholds used). Then, once a linkage strategy has been implemented, we can estimate linkage error rates to help us understand whether and how linkage error might impact on analysis results. Available techniques for assessing rates and distributions of linkage error are discussed in Results, using examples from published literature.

### Methods for handling linkage error in analysis

Development of methods to handle bias due to linkage error has been identified as a priority for research (Jorm 2015; Wellcome’s Longitudinal Population Studies Working Group 2017). There are several practical approaches that can be taken, including statistical adjustments based on estimated error rates and distributions (quantitative bias analysis) and probabilistic techniques involving imputation or weighting. Quantitative bias analysis aims to address the sensitivity of the analysis to underlying assumptions about linkage error by estimating the potential impacts of linkage error in terms of misclassification, measurement error and selection bias. Probabilistic techniques go one step further, by attempting to reflect *uncertainty* in linkage, as well as bias. These approaches are discussed further in the following sections.

## Results

### Assessing linkage quality in cohort studies

A range of methods aiming to assess linkage quality can be found in the literature, and examples of these are provided in Table 3. Which of these methods is possible to implement will depend on the linkage structure (e.g. whether we expect all records in one file to have a match), and on the level of access to data or collaboration with data linkers. Often, linkage is performed by a trusted third party, meaning that analysts do not have access to identifiers, and data linkers do not have access to any attribute data (e.g. clinical variables, survey responses, etc.) (Kelman et al. 2002). Table 3 outlines the requirements of access to identifiers or de-identified data for each method: the first three techniques can only be implemented by those with access to identifiers but the remaining techniques can be employed by analysts of de-identified linked data, provided that information about the quality of links and identifiers is provided by data linkers (Paixão et al. 2019).

**Table 3.** Methods for estimating rates and distributions of linkage error.

Method	Description	Example	Requirements
Manual review	Manual inspection of record pairs is used to make a decision about whether two records belong to the same individual or not, based on similarities of identifiers held in those records. Humans may recognise small differences between identifiers that may not have been fully captured in an automated linkage strategy (e.g. recognising that Beth is a derivative of Elizabeth, or that December 31 1999 is close to January 01 2000).	Manual review is routinely use at the Centre for Health Record Linkage (CHeReL; New South Wales Ministry of Health). (Centre for Health Record Linkage 2012). CHeRel hold linked records from a number of administrative datasets, including records of hospitalisations, emergency department presentations, births, cancer registrations and deaths. Manual review of a subsample of the 114 Million Brazil cohort has been used to generate training data to inform machine learning approaches to assessing linkage quality (Pita et al. 2017).	Access to identifiers
Applying a linkage algorithm to a subset of (gold-standard) data	Testing a linkage strategy on a sample of data where the true match status is known can provide estimates of linkage error rates. “Gold-standard” or “training” datasets might come from a subset of data where a unique identifier is available, where manual review can be performed on a sample of data, or where external information is available. If a subsample is used, it should be representative of the quality of the main dataset.	Linkage of admission records for children in intensive care with laboratory records from infection surveillance systems. In this study, 2 of the 22 laboratories were able to provide high quality, complete and unique identifiers that were used to create a gold-standard subsample. (Harron et al. 2013).	Access to identifiers within a gold-standard or training dataset
Applying a linkage algorithm to “negative controls”	Testing a linkage strategy on a subset of data we are sure should not link (i.e. data from two unrelated populations) can be a convenient way of identifying false match rates.	Linking birth records to hospital records for pregnant women known to have had an abortive outcome (i.e. where no birth record should exist). (Paixão et al. 2019).	Access to identifiers
Identification of implausible scenarios	False matches can be identified in cases where (non-identifiable) information in the records mean it is unlikely that two records belong to the same individual (e.g. a male patient being admitted for a caesarean section, or an admission following a death). In cases where we expect there to be a maximum of one match per record (e.g 1:1 or many:1 matching), multiple matches per record will indicate one or more false matches. Identifying false matches in these ways can provide a minimal estimate of the false match rate.*	Identifying false matches through implausible sequences of events in hospital data, e.g. multiple admissions on the same day in different parts of the country.(Hagger-Johnson et al. 2015) Estimating the number of false matches by counting the number of duplicate matches between Census and mortality records. (Blakely and Salmond 2002).	Access to attribute data and knowledge about potential implausible scenarios
Comparison of linked and unlinked records	In a “Master” or “Nested” structure where we expect all cohort records to link, the number of missed matches can be estimated as the number of records that failed to link. In other linkage structures, sometimes a known subset of cohort members will be expected to link (“positive controls”), for example when information about disease or vital status can be obtained from other data sources as well as linkage to patient/death registers. In these cases, linked and unlinked members of the subset can be compared.**	Comparing the characteristics of linked and unlinked maternal and baby hospital records in New South Wales.(Ford et al. 2006) Linkage of a subset of prisoners known to have died in prison: after linkage to a register of deaths, the match rate among this subgroup was assessed and the characteristics of linked and unlinked records in this subset could have been compared. (Moore et al. 2014).	Access to unlinked records with attribute data
Comparison of records with high versus low quality identifiers	Records with missing or invalid identifiers may be less likely or even impossible to link in many applications, so comparing the distribution of identifier quality with respect to variables of interest can provide information about the minimum number of missed links (those with insufficient data for linkage) and the likely distribution of missed links with respect to variables of interest.	Comparison of records with and without a valid NHS number for linkage of tuberculosis case notifications and a laboratory database of all culture positive isolates from tuberculosis reference laboratories. (Aldridge et al. 2015).	Access to record-level or aggregate indicators of identifier quality and attribute data
Comparisons with external data sources	In situations where the expected number of links is not known *a priori*, comparing the characteristics of a linked sample of records with other representative data can help identify whether the linked records are broadly representative, or whether linkage errors might have contributed to observed differences.	Comparing the characteristics of a cohort of linked mother-baby records with national published statistics on birth characteristics. (Harron et al. 2016).	Access to attribute data only

* More false matches might be present, but unidentified.

** Technically, if there are false matches, there will also be additional missed matches (i.e. if a link is made to the wrong record, it will not appear as a missed match, but we will have missed the correct match).

### Handling linkage error in analysis: quantitative bias analysis

A simple approach to understanding the impact of linkage error in analysis is to consider the best- and worst-case scenarios: how much of each type of linkage error *could* there be, and how strongly might the error be correlated with variables of interest? This type of quantitative bias analysis can be sufficient to demonstrate the sensitivity of results to the range of plausible assumptions that could be made about linkage error. For example, based on a linked electronic cohort of children with Down’s syndrome, we specified plausible ranges for a set of “bias parameters” that were relevant to a given analysis, including the numbers of missed matches and false matches, and the proportion of false matches that occurred between comparable records (Doidge et al. 2019). We then tested the robustness of our results to different specifications of these parameters.

Wherever possible, it is best to inform assumptions about plausible ranges of error rates with formal assessments of linkage quality. Moore et al. demonstrate one such example, in which a cohort of prisoners was linked to a register of deaths to compare mortality rates between prisoners with and without psychiatric hospitalisations (Moore et al. 2014). Links with mortality records were meaningfully interpreted as implying the vital status (dead or alive) of prisoners, so missed matches would be expected to lead to false negative misclassification of death. False matches would be expected to lead to false positive misclassification if occurring with a person who was alive, but not if they occurred in people who had died. It would be possible to adjust mortality rates if information were available about the rate of missed matches, and the rate and distribution of false matches with respect to vital status (i.e. the rates of false matches among people who were alive and dead, respectively).

Although the expected number of matches was not known *a priori*, two useful subsets of records with known match status were identified: people who were known to be still in prison at the observational endpoint of the available death registrations and therefore should not link (“negative controls”), and people who were known to have died in prison and therefore should link (“positive controls”). By examining match rates among the positive controls, the authors were able to estimate the sensitivity of survival classification (the proportion of cohort deaths linked) (Table 4). By examining match rates among the negative controls, they could estimate the specificity of survival classification (proportion of living cohort linked, i.e. false matches). The authors use these to adjust estimates of mortality accordingly and, by applying the same estimates of sensitivity and specificity to patients with and without psychiatric hospitalisations, demonstrate that even in the absence of differential linkage error, the relative risk of death was still biased towards no association. Ideally, estimates of sensitivity and specificity would have been produced separately for each subgroup, which could have resulted in different adjustments.

**Table 4.** Quantitative bias analysis for linkage error in a cohort linked to a register of deaths.

	Subgroups with known vital status	
	Dead (“positive control”)	Alive (“negative control”)
Linked	275	23
Not linked	36	7535
Sensitivity of survival classification	275/(275 + 36) = 0.884	
Specificity of survival classification	7535/(7535 + 23) = 0.997	

Data reproduced from Moore et al. (2014). Note that sensitivity and specificity of classification are not equivalent to the sensitivity and specificity of linkage, which is unknown. Also note that, because the positive controls and negative controls would not be expected to form a representative sample of the cohort, it is not possible to calculate positive or negative predictive values from this table (i.e. rows cannot be summed).

### Handling linkage error in analysis: probabilistic analysis

In complex linkage scenarios, or where there are multiple variables of interest, estimating linkage error rates across subgroups may not be straightforward. In these situations, imputation-based methods can provide a useful approach to handling linkage error and representing uncertainty in linkage. In generating one version of a linked dataset in the presence of imperfect identifiers, errors will be inherent; different versions of a linked dataset could be constructed according to how data are pre-processed, how linkage is conducted, and how decisions about thresholds are made. Imputation based approaches re-frame linkage as a missing data problem, and the aim moves away from identifying definite links between records, to carrying through the correct values into analysis, along with appropriate measures of uncertainty.

Consider a “Nested” design in which we expect all records in one file to link (usually the cohort), but records with missing data are excluded from analysis. In this setting, the problem is analogous to complete case analysis (discussed in detail in the missing data literature, see for e.g. (Sterne et al. 2009)), where biases may be introduced depending on the missingness (or linkage) mechanism. Appropriate use of strategies to address missing data (e.g. multiple imputation) might therefore mitigate the impact of linkage error in this scenario. Information on the association between covariates and variables derived through linkage can be obtained from certain links and certain non-links. This information can then be used in an imputation model, to impute variables that were not possible to derive with certainty through linkage. Estimates of quantities of interest are then combined over a number of imputed datasets, using Rubin’s rules for multiple imputation (Rubin 1987).

Multiple imputation has been shown to be an effective approach for handling linkage error, and specifically missed matches (Zhang et al. 2016). However, the procedure described above ignores information that we do have about the potential values of variables derived through linkage (i.e. from the candidate linking records). For example, in Table 5 we present linkage of five cohort records with cancer registry records. For three of these records, we are certain about whether there is a link or not. For the remaining two records, there is some uncertainty about whether there should be a link. We could treat cancer status for these uncertain links as missing and impute according to standard multiple imputation methods (e.g. based on the available information on sex, age, SES and any other predictor variables). However, the uncertainty about whether or not there should be a link is represented by the associated match weight for each candidate linking record. These values are therefore not entirely missing, but “partially observed” (Goldstein et al. 2009). Information on the potential values that these variables could take is given by candidate records and associated match weights.

**Table 5.** Imputation-based approaches for handling linkage error in analysis: an example based on 5 cohort records linked with cancer registry records with varying levels of certainty.

	Sex	Age	SES	Cancer	Linkage certainty
1	Male	55	Low	Yes	Certain link
2	Male	45	High	Yes	Certain link
3	Female	46	High	No	Certain non-link
4	Male	48	Low	?	Match weight = 15
5	Female	52	High	?	Match weight = 2

Records 1–3 are considered to have complete data; records 4 and 5 are considered to have missing or partially observed data. In multiple imputation, missing data for records 4 and 5 would be imputed based on the observed characteristics (sex, age, SES) and the relationship between these characteristics and the outcome (Cancer) in the complete records. In prior-informed imputation, the posterior distribution for the imputation would be informed, in addition, by the match weights in the candidate linking records (i.e. match weight = 15 for record 4 would provide more evidence of a match than match weight = 2 for record 5).

We can therefore form a probability distribution that is a direct function of the set of match weights for each uncertain record (records 4 and 5 in Table 5). This probability distribution forms a prior distribution for the variable of interest (in this case, cancer status). The prior distribution is then combined with the (conditional) likelihood for the variables of interest based on the certain linked records (records 1–3 in Table 5) to form an updated posterior distribution. Values can then be sampled from the posterior distribution based on the standard multiple imputation framework (Goldstein et al. 2012).

A number of imputed datasets are again produced, with estimates and standard errors averaged over the imputed data (Rubin 1987). This method, known as “prior-informed imputation” has been shown to be effective at avoiding bias due to linkage error in some settings, and for providing standard errors that properly account for the uncertainty arising from linkage error (Harron et al. 2014). Prior-informed imputation is particularly useful for handling differential linkage according to subgroups, as this is accounted for within the imputation.

## Discussion

In this paper, we outline some of the challenges in using data linkage to enhance or create longitudinal cohort studies. We discuss the mechanisms by which linkage error can introduce bias into results, describe a number of methods that are available for estimating linkage error rates, and illustrate two approaches for incorporating information on linkage quality into analysis. In maximising the potential of linked data in cohort studies, the relationship and communication between data providers, linkers, and analysts is key.

Firstly, an iterative process can be used to develop a linkage strategy that is tailored towards a particular research question or design (including a discussion of trade-offs between sensitivity and specificity of linkage, if necessary). This should include input from both those who know how the data have been generated, and those who know how the linked data will be analysed. An iterative process, where initial linked datasets are created and evaluated, allows analysts to feedback information about any implausible links, and to understand the balance between false and missed matches.

Secondly, it is important to retain as much information about the linkage process as possible. If deterministic linkage has been used, then a match rank, or description of the linkage step achieved (i.e. an agreement pattern for a set of known identifiers), can be provided alongside each record pair. In probabilistic linkage, match weights can be provided for each record pair. It is also helpful to provide information on multiple candidate links, especially where there is a small margin of certainty about which is the most likely match. This allows the researcher both to perform quality assurance (i.e. ensuring that the highest weighted record really is the best match, based on any other available data) and to incorporate this uncertainty into analysis (e.g. using imputation-based approaches as described above). Methods and software to handle linkage error within analysis are currently being developed under a Wellcome Trust grant (212953/Z/18/Z). Guidelines are available to provide advice on the information that can and should be shared between data providers, data linkers and data analysts, in order to facilitate careful evaluation of linkage quality (Gilbert et al. 2018).

Linkage error is one aspect of data quality that should be considered when using administrative data for research, when these data were not collected primarily for research purposes. The expansion of research using administrative data makes it challenging to identify, *a priori*, the specific challenges and potential for bias that might be important for a given dataset or analysis. The relative importance of linkage errors should therefore be considered in the context of other potential causes of bias including inaccurate or incomplete recording or coding of exposures and outcomes (Benchimol et al. 2015).

Linkage of administrative data holds great potential for maximising the value of existing cohort studies, and for generating new electronic cohorts on a larger and more detailed scale than has previously been possible. When conducted carefully, linkage can generate data that are less subject to attrition or response bias, in a more efficient and cost-effective manner that poses less burden on participants. However, a lack of unique identifiers for linkage means that linkage strategies need to be developed with care. Methods that provide a quantitative approach to classifying record pairs (i.e. using match weights) can help researchers understand the trade-off between false and missed matches. Regardless of the linkage methods used, some errors are likely to remain, reflecting the dynamic and imperfect nature of administrative data that are generated as individuals interact with different services over time. It is vital that the mechanisms by which these errors might impact on results are considered, so that potential biases can be identified and mitigated in analysis.
